# An old friend with new skills: Imiquimod as novel inhibitor of Hedgehog signaling in basal cell carcinoma

**DOI:** 10.18632/oncoscience.80

**Published:** 2014-09-16

**Authors:** Wolfgang Gruber, Anna-Maria Frischauf, Fritz Aberger

**Affiliations:** ^1^ Department of Molecular Biology, Division of Molecular Tumor Biology, University of Salzburg, Salzburg, Austria

**Keywords:** Hedgehog signaling, Imiquimod, GLI proteins, basal cell carcinoma, Adenosine receptors, ADORA, Protein Kinase A, PKA

## Abstract

Deregulated Hedgehog (HH)/GLI signaling plays an etiologic role in the initiation, progression and maintenance of many cancers. Small molecule targeting of HH signaling by inhibiting the essential pathway effector Smoothened (SMO) has proven exceptionally efficient for the treatment of advanced and metastatic basal cell carcinoma. That said, severe side effects, limited response rates, SMO-independent GLI signaling and rapid development of drug resistance limit the therapeutic success of SMO antagonists, urgently calling for the identification of alternative and additional strategies repressing oncogenic HH signaling.

In this perspective article we highlight recent findings showing that the Toll-like receptor-7/8 (TLR7/8) agonist imiquimod (IMQ), an immune modulator approved for the treatment of basal cell carcinoma, can also act as a potent cell autonomous inhibitor of oncogenic HH signaling. Surprisingly, IMQ reduces HH signal strength independent of TLR signaling, via adenosine receptor (ADORA)/Adenylate cyclase (AC)/Protein kinase A (PKA) activation. We here highlight the molecular mechanisms of IMQ-mediated repression of HH/GLI and discuss the possible benefits as well as challenges of using ADORA agonists for the treatment of HH-associated cancer.

## INTRODUCTION

HH/GLI signaling is crucial for proper embryonic development and in adults for tissue maintenance and regeneration by regulating stem cell activation and self-renewal. In line with the requirement of exquisite regulation of signal strength and duration, deregulated HH/GLI signaling can have fatal consequences causing developmental anomalies and cancer (for extensive reviews see [1-11]).

**Figure 1:**
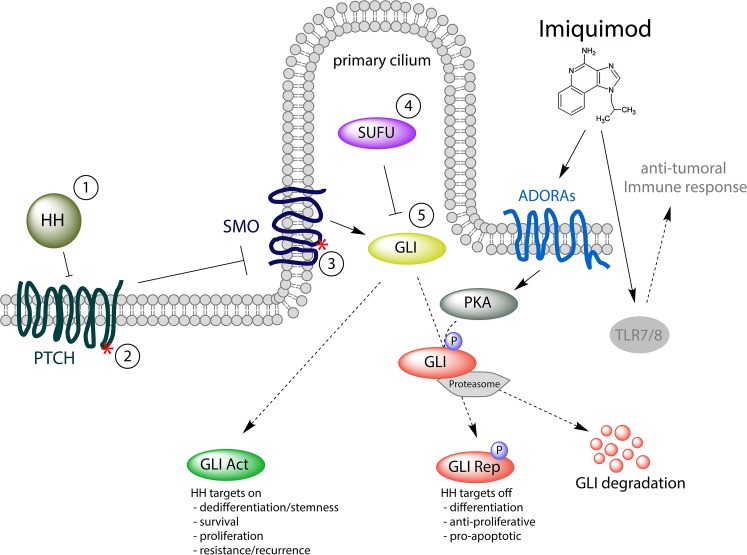
IMQ represses HH/GLI signaling via ADORA/PKA activation Classical Hedgehog (HH) signaling is activated by secreted HH protein binding to its receptor PTCH (1). In cancer, loss of function mutations in *PTCH* (2), activating mutations in *SMO* (3), genetic loss of *SUFU* (4) or *GLI1/2* amplification/overexpression (5) result in aberrant HH signaling and an increased GLI activator (GLI Act) to GLI repressor (GLI Rep) ratio, inducing HH target gene expression (e.g. GLI1, HHIP) and oncogenic transformation. Imiquimod (IMQ) activates Protein Kinase A (PKA) by engaging adenosine receptors (ADORAs), leading to GLI phosphorylation and functional inactivation via proteasome-mediated GLI repressor formation and/or GLI degradation. We propose that IMQ can block HH signaling in all pathway-activating events illustrated, even in settings where SMO inhibitors may no longer be effective (i.e. in settings 4 and 5, where GLI activation occurs in a SMO-independent manner, including GLI activation by other oncogenic pathways, for details see main text).

HH pathway activation is initiated by binding of HH ligand to Patched (PTCH), a twelve transmembrane domain protein that blocks Smoothened (SMO) in the absence of HH. Ligand binding inhibits the repressive function of PTCH, allowing SMO to enter the primary cilium, an antenna-like organelle essential for HH signal coordination and transduction [12-17]. Ciliary activated SMO subsequently activates GLI2/3 transcription factors, which represent the downstream effectors of canonical HH signaling (Figure 1). HH/GLI target genes include feedback signaling proteins (e.g. PTCH, HHIP and GLI1) and genes involved in proliferation/cell cycle regulation, differentiation, self-renewal/stemness, metastasis and survival (for reviews see [3, 6, 18-20]). Suppressor of Fused (SUFU), a key negative regulator of mammalian HH signaling, directly binds to GLIs, thereby preventing their activation and nuclear localization [21]. In addition, proteolytic processing yielding C-terminally truncated GLI repressor forms and proteasome-dependent degradation of GLI proteins constitute critical negative regulatory mechanisms. In this context, phosphorylation of GLIs by protein kinase A (PKA) is a key repressive step in HH/GLI signaling that promotes GLI repressor formation and GLI destabilization [22-36].

Of note, GLI transcription factors can be activated also in a non-canonical SMO independent manner, thereby reversing the therapeutic effect of SMO inhibitors used for targeted inhibition of oncogenic HH signaling [37-52].

The oncogenic role of HH signaling in cancer was first discovered in patients suffering from nevoid basal cell carcinoma syndrome (NBCCS) caused by genetic loss of PTCH function. NBCCS patients are prone to developing multiple basal cell carcinomas (BCC) in response to ligand independent constitutive activation of the HH pathway [53-56]. Clinical trials with the first FDA approved HH pathway inhibitor vismodegib (Erivedge), a selective SMO inhibitor, showed that targeting HH in BCC patients dramatically reduces tumor burden and prevents growth of new lesions [57-61]. However, more than 50% of patients receiving vismodegib discontinued drug treatment due to severe side effects including muscle cramps, nausea, hair, taste and weight loss [58, 59]. The efficacy of SMO inhibitors can be further limited by rapid development of drug resistance via mutations in SMO, genetic alterations downstream of SMO (e.g. loss of SUFU or gain of GLI copy number) or by the activation of compensatory pathways such as PI3K/AKT [50, 62-65].

The immune modulator imiquimod (IMQ, applied as 5% cream formulation referred to as Aldara) represents another FDA approved drug successfully applied for the treatment of superficial BCC, when surgery is less favorable [66-70].

IMQ is a synthetic nucleoside analogue of the imidazoquinoline family [71]. Its anti-tumor activity is multifactorial and not completely understood. IMQ is known to bind to and activate Toll-like receptors 7/8 (TLR7/8) thus stimulating TLR-MYD88 signaling. The resulting inflammatory reaction and antitumor response involves plasmacytoid dendritic and cytotoxic CD8+ cells attacking the tumor [72-74]. A direct effect of IMQ on oncogenic HH/GLI signaling in BCC has not been reported until recently.

In a screen for modifiers of HH/GLI signaling that comprised several TLR agonists including IMQ, our group noticed that IMQ has a direct repressive effect on GLI activity in mouse embryonic fibroblasts (H. Esterbauer, personal communication and unpublished data). In light of the well-documented therapeutic effect on BCC, this led us to hypothesize that IMQ may directly repress oncogenic HH/GLI signaling independent of its immune modulating function.

In the study by Wolff et al. [75], we tested for a putative direct effect of IMQ on HH signaling and found that IMQ directly blocks HH pathway activation in cultured murine BCC cells as evidenced by the repression of HH target genes including Gli1. Surprisingly, BCC cells do not express detectable levels of the cognate IMQ receptors TLR7/8, neither did genetic inhibition of the essential TLR effector MYD88 affect the repressive activity of IMQ on HH/GLI signaling. This suggested a non-classical, TLR-MYD88 independent effect of IMQ on HH/GLI signaling.

Two previous studies were key to interpret these unexpected and puzzling findings. Schön et al. have shown that IMQ can affect adenylate cyclase (AC) and protein kinase A (PKA) activity via binding to adenosine receptors (ADORAs) independent of TLR7/8 [76]. Equally important, a study analyzing hematopoietic progenitors in flies has identified adenosine/ADORA signaling as a negative regulator of Hh signaling via activation of PKA and repression of the fly GLI homologue Cubitus interruptus [77].

In line with these data, we observed that treatment of BCC cells or human GLI expressing keratinocytes with IMQ induced PKA-mediated GLI phosphorylation, thereby reducing the level of GLI activator and oncogenic HH signal strength, respectively (Figure 1).

The study by Wolff et al. therefore identified ADORAs as possible targets for inhibition of HH signaling in BCC, and it is tempting to speculate that other small molecule ADORA agonists currently in clinical or preclinical evaluation may also hold promise for anti-cancer therapy by repressing HH/GLI signaling. In this context it will also be important to address whether ADORA agonists can overcome current limitations of SMO inhibitors for the treatment of acquired or de novo SMO inhibitor resistant malignancies including cancers with SMO-independent GLI activation [9, 46, 48, 50, 63-65].

## OPEN QUESTIONS AND FUTURE CHALLENGES

Although the surprising finding of a direct repressive role of IMQ on HH/GLI via stimulation of ADORA/AC/ PKA signaling revealed a new mode of action of a well-known drug, several questions remain to be addressed before any of these findings may be translated into clinical applications:

### (1) Which ADORA subtype is responsible for the cell autonomous effect of IMQ?

In Wolff et al. [75] we investigated the expression of ADORA subtypes in human BCCs and demonstrated that ADORA2A and ADORA3 are overexpressed in BCCs compared to normal skin, whereas expression of ADORA1 and ADORA2B was comparable to normal skin samples [75]. By applying selective ADORA2A agonists and antagonists [78, 79] we showed that ADORA2A has a key role in mediating the HH-repressive effects of IMQ. However, quantification of GLI2 phosphorylation showed stronger phosphorylation by IMQ than by ADORA2A agonists. This may be explained by an additional receptor-independent activation of PKA by IMQ or by an additive effect resulting from activation of multiple ADORA subtypes by IMQ [76]. Subtype specific knockdown of each of the four ADORAs alone or in combination will therefore be important to understand the individual contribution of the respective ADORA family members to HH signal repression.

### (2) Do distinct cancer entities engage different ADORA subtypes in the modulation of HH/GLI signaling?

A number of HH-associated cancer entities such as cancers of the skin, breast, lung and prostate express high levels of different ADORA subtypes [9, 80]. While the study by Wolff et al. suggests that ADORA2A may be the main negative regulator of HH signaling in BCC, it is well possible that other ADORA subtypes negatively or even positively modulate HH signaling in cancer entities other than BCC. The regulatory complexity is likely to be very high given the fact that ADORA signaling can also act as inducer of extracellular signal-regulated kinase (ERK) 1/2 activity [81, 82]. As ERK1/2 activation is a potent stimulus and modifier of GLI activity [39, 41, 44, 45, 83], ADORA signaling may also positively affect HH/ GLI signal strength in a context dependent manner. The use of IMQ as potential therapeutic for HH/GLI associated cancers therefore needs to be carefully evaluated for each tumor entity.

### (3) Where does IMQ interact with HH/GLI signaling?

Detailed epistatic mapping of the repressive mechanism of IMQ on HH/GLI signaling will be key to stratify patients with HH-associated cancer into putative responders and non-responders. The data by Wolff et al. support a model where IMQ interferes with HH signaling downstream of SMO (Figure 1), suggesting that ADORA agonists may prove beneficial also for SMO-inhibitor resistant and SMO-independent cancer entities. This would also be an indication that combination treatments with vismodegib and ADORA agonists may improve therapeutic efficacy and possibly also prevent or at least delay the development of drug resistance. For this, additional experiments need to be performed to evaluate the efficacy of IMQ in cancer cells expressing drug-resistant variants of SMO or lacking SUFU, the key negative regulator of GLI.

The identification of IMQ as inhibitor of HH/GLI signaling has strong potential to broaden the spectrum of applications for this drug or derivatives thereof. However, addressing the open questions raised in this article is critical and there is still a significant way to go before optimized ADORA agonists can be clinically evaluated as potential drugs for the treatment of HH/GLI dependent cancers.
